# Usability of detecting delivery errors during treatment of prostate VMAT with a gantry‐mounted transmission detector

**DOI:** 10.1002/acm2.13260

**Published:** 2021-05-05

**Authors:** Hirofumi Honda, Masahide Tominaga, Motoharu Sasaki, Masataka Oita, Hiromitsu Kanzaki, Yasushi Hamamoto, Yoshiaki Ishii, Ryuji Yamamoto, Teruhito Mochizuki, Teruhito Kido, Yoshihiro Uto

**Affiliations:** ^1^ Department of Radiological Technology Ehime University Hospital Ehime Japan; ^2^ Graduate School of Advanced Technology and Science Tokushima University Tokushima Japan; ^3^ Institute of Biomedical Sciences Tokushima University Graduate School Tokushima Japan; ^4^ Okayama University Graduate School of Interdisciplinary Science and Engineering in Health Systems Okayama Japan; ^5^ Department of Radiation Therapy National Hospital Organization Shikoku Cancer Center Matsuyama Japan; ^6^ Department of Radiology Ehime University School of Medicine Ehime Japan; ^7^ Graduate School of Technology, Industrial and Social Science Tokushima University Tokushima Japan

**Keywords:** prostate, transmission detector, volumetric‐modulated arc therapy

## Abstract

Volumetric‐modulated arc therapy (VMAT) requires highly accurate control of multileaf collimator (MLC) movement, rotation speed of linear accelerator gantry, and monitor units during irradiation. Pretreatment validation and monitoring of these factors during irradiation are necessary for appropriate VMAT treatment. Recently, a gantry mounted transmission detector “Delta^4^ Discover® (D4D)” was developed to detect errors in delivering doses and dose distribution immediately after treatment. In this study, the performance of D4D was evaluated. Simulation plans, in which the MLC position was displaced by 0.5, 1.0, 1.5, 2.0, 2.5, and 3.0 mm from the clinically used original plans, were created for ten patients who received VMAT treatment for prostate cancer. Dose deviation (DD), distance‐to‐agreement (DTA), and gamma index analysis (GA) for each plan were evaluated by D4D. These results were compared to the results (DD, DTA and GA) measured by Delta^4^ Phantom + (D4P). We compared the deviations between the planned and measured values of the MLC stop positions A‐side and B‐side in five clinical cases of prostate VMAT during treatment and measured the GA values. For D4D, when the acceptable errors for DD, DTA, and GA were determined to be ≤3%, ≤2 mm, and ≤3%/2 mm, respectively, the minimum detectable errors in the MLC position were 2.0, 1.5, and 1.5 mm based on DD, DTA, and GA respectively. The corresponding minimum detectable MLC position errors were 2.0, 1.0, and 1.5 mm, respectively, for D4P. The deviation between the planned and measured position of MLC stopping point of prostate VMAT during treatment was stable at an average of −0.09 ± 0.05 mm, and all GA values were above 99.86%. In terms of delivering doses and dose distribution of VMAT, error detectability of D4D was comparable to that of D4P. The transmission‐type detector “D4D” is thus suitable for detecting delivery errors during irradiation.

## 1 INTRODUCTION

Currently, volumetric‐modulated arc therapy (VMAT), is being widely performed.^1^, ^2^ VMAT requires highly accurate control of multileaf collimator (MLC), rotation speed of linear accelerator gantry, and delivering monitor units during irradiation. Even small errors in these factors would lead to significant accidents in highly precise radiotherapy such as VMAT; therefore, patient‐specific pretreatment verification of MLC movement and gantry rotation speed are necessary for VMAT.^3^, ^4^ In addition, there exists a possibility of failure of the linear accelerator or its control system while the patient is being treated. Some serious accidents have already occurred in clinical radiotherapy.^5^, ^6^, ^7^, ^8^, ^9^ In one of these accidents, MLC opened incorrectly during intensity modulated radiotherapy for head and neck cancer, and the patient was seriously injured.^9^ Owing to a hang‐up while using the treatment planning system(TPS), the MLC control point data, which should have been present in the treatment planning data, were absent.^9^ The accident occurred when a large number of monitor units were delivered to patients without the MLC control point data.^9^ Therefore, prior patient‐specific verification is imperative to prevent such an accident.^3^, ^4^ However, according to a report from the Netherlands in 2010, dose errors could not be detected even after patient‐specific preverification, and they were finally detected by in vivo dosimetry (IVD) using an electronic portal imaging device (EPID) during treatment.^10^, ^11^ Therefore, not only pretreatment verification but also monitoring of delivering doses and dose distribution during irradiation are necessary for an efficient performance of the VMAT.^12^ In recent years, the importance of dose verification during irradiation has been recognized.^10^, ^11^ Accordingly, EPID‐based IVD is expected to be used for dose verification during irradiation.^13^ In traditional IVD, thermoluminescent dosimeter and diode detector placed on the surface of the patient’s body have been used for dose verification during irradiation.^13^ However, these dosimeters do not seem to be suitable for IVD of VMAT that requires complex dose distribution and steep dose gradient.^13^ Furthermore, real‐time dose monitoring is impossible for IVD using EPID because IVD using EPID requires a recalculation of the doses monitored during treatment. For the precise detection of delivery errors during irradiation in VMAT, a gantry‐mounted transmission detector was developed. Gantry‐mounted transmission detectors allow monitoring of delivering doses and dose distribution during irradiation immediately after treatment. Errors can be automatically detected immediately after treatment by comparing the dose monitored by the transmitting detector with the planned dose. Therefore, these systems have an advantage in the detection of delivery errors during irradiation dose monitoring and have been applied clinically.^14^, ^15^

The purpose of this research is to investigate the possibility of detecting errors during treatment by performing basic experiments that create an error plan for the stop position of MLC using a gantry‐mounted transmission detector. This study was conducted based on the assumption that a systematic error occurred owing to the installation position error of the leaf offset during MLC adjustment. In this study, we compared the error detection capabilities of the gantry‐mounted transmission detector used for monitoring during treatment and a three‐dimensional detector used for pretreatment patient quality assurance (QA). If the error detection capabilities of both the detectors are verified to be comparable, the gantry‐mounted transmission detectors can be used independently and will be useful in clinical practice.

## 2 MATERIALS AND METHODS

### 2.A Material

In this study, we used the Delta^4^ Discover (D4D) system (ScandiDos AB, Uppsala, Sweden) as the monitoring system to detect errors in dose and MLC position during treatment as well as delivery errors during irradiation. This system consists of Delta^4^ Phantom + (D4P) (ScandiDos AB, Uppsala, Sweden) used for patient‐specific preverification and the D4D gantry‐mounted transmission detector.

The linear accelerator used was a TrueBeam (Millennium 120 MLC 5 mm leaf) (Varian Medical Systems, California, USA), and 10X energy was applied. To ensure the output stability, the output coefficient of variation of the output measurement of weekly QA during the data collection period was calculated. The coefficient of variation of the output dose was found to be stable at 0.19% in the weekly QA dose control using the ionization chamber. The calculated dose of TPS was obtained using Eclipse version 11.0 (Varian Medical Systems, California, USA) based on the anisotropic analysis algorithm.

#### 2.A.1 Delta^4^ discover

The very thin, disk‐shaped main unit with a diameter of 790 mm and a thickness of approximately 22 mm measured from the front of the collimator, was designed to fit inside the TrueBeam laser guard. The D4D consisted of 4,040 p‐type diode detectors with a diameter of 1.0 mm and a thickness of 0.1 mm. Regarding the detector arrangement, the elements were separated by approximately 1.5 mm, in the X direction (parallel direction along the MLC trajectory) and by 3.0 mm approximately in the Y direction (direction perpendicular to the MLC trajectory). This arrangement is equivalent to 2.5 mm and 5.0 mm intervals in the X and Y directions, respectively, converted to the isocenter plane; the detector covered 195 × 250 mm on the isocenter. The overall thickness, including the detector cover, was 5.5 cm, and the source‐to‐detector distance was 604 mm. In addition, as a transmission type detector, it has a small effect on the beam transmission and surface dose.^16^

#### 2.A.2 Delta^4^ phantom +

D4P consists of 1,069 diodes on two orthogonal boards and a p‐type diode detector with the same 1.0 mm diameter as in D4D. In the central area (60 mm × 60 mm), the detectors are placed at 5 mm intervals, while on the periphery, they are at placed 10 mm intervals. They cover a total area of 200 mm × 200 mm. These detectors were cased inside an acrylic cylindrical phantom that had a diameter of 22 cm and length of 40 cm.^17^

### 2.B Treatment planning

#### 2.B.1 Original plan

In this study, new plans (original) for evaluation were created based on the VMAT plans used in clinical practice for ten prostate cancer patients (2‐arc plan with 181°–179° clockwise (CW) rotation and 179°–181° counterclockwise (CCW) rotation). The original plans were changed to 1‐arc plans (181°–179° CW rotation) without changing the optimization parameters. The collimator angle was 30°. The number of control points of MLC of the plan created by the treatment planning device was 178.

#### 2.B.2 Simulated plan for detecting errors during treatment

**Fig. 1:**
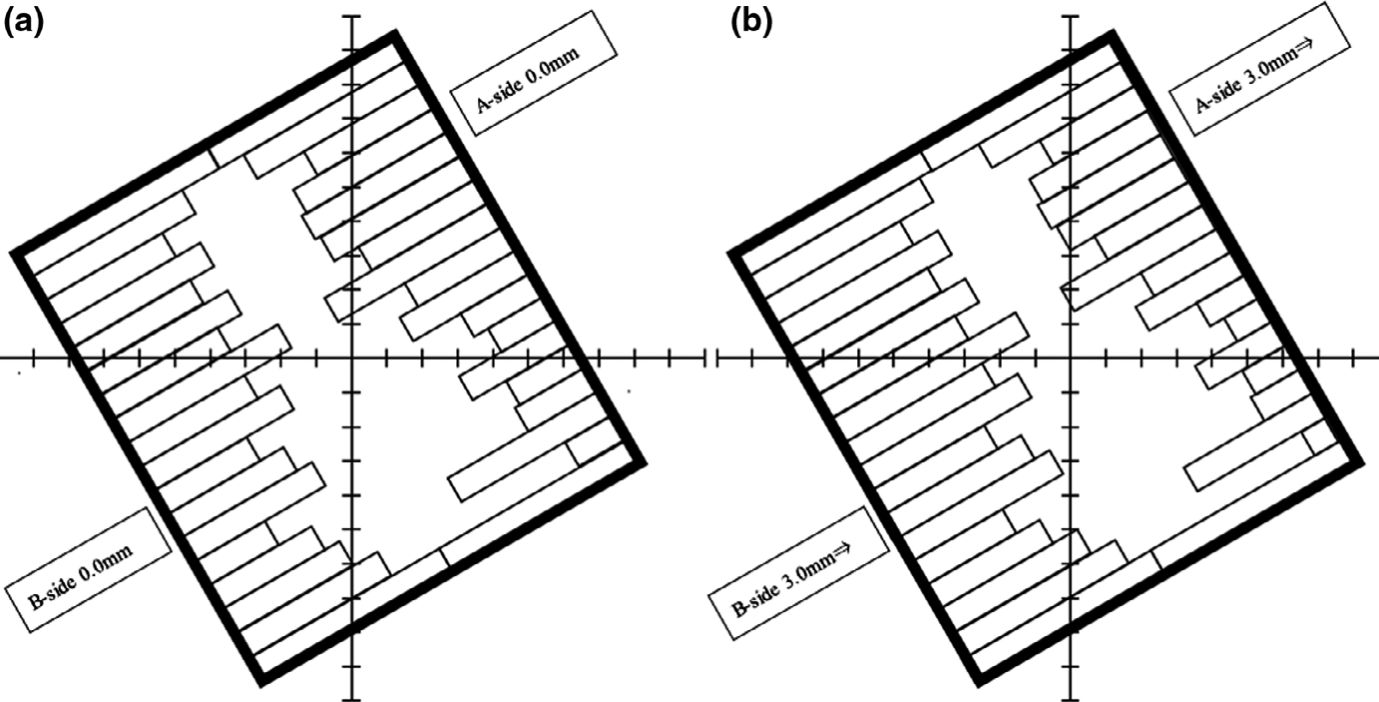
Schematics of MLC same‐direction sifts. (a) Schematic of the original MLC position. (b) Schematic of the stop position of A‐side and B‐side MLC displaced 3.0 mm from B‐side to A‐side direction.

Six types of errors were intentionally introduced to the original plans considered in this study. The simulation plan for detecting errors during treatment consisted of the MLC of all the control points displaced from the B‐side (X1 side) to the A‐side (X2 side) by 0.5, 1.0, 1.5, 2.0, 2.5, and 3.0 mm from the original plan. Figure 1 shows a schematic diagram of the MLC stop position in the original plan and the MLC stop position where the MLC stop positions on the A‐side and B‐sides are displaced by 3.0 mm.

### 2.C Method

**Fig. 2:**
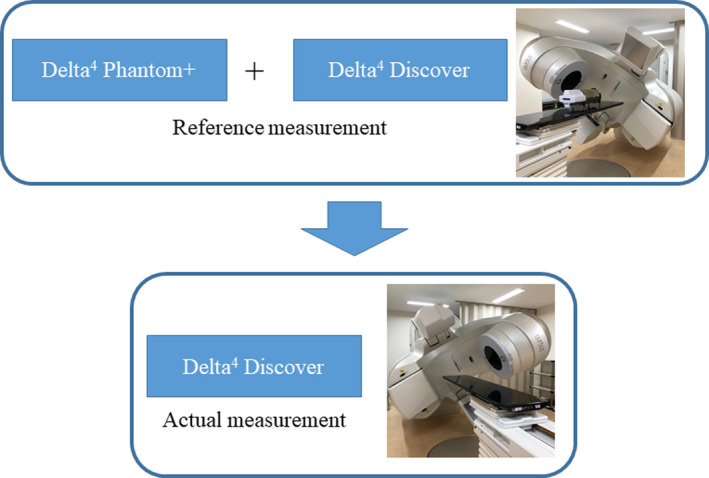
Procedure of D4D measurement. Combine D4P and D4D to measure the reference measurement using the "synthetic mode". Next, the fluences are converted into doses by directly measuring using D4D. * Reference measurement of this process should be performed for all dose verification cases.

First, the D4P was setup at the isocenter using the treatment room laser. Next, 100MU irradiation was performed from a gantry angle of 0° and 90° in a 10 cm × 10 cm irradiation field, and the profile was confirmed using the software provided with D4P. Based on the profile calculated by TPS, the TrueBeam couch was moved in 0.1 mm increments to a position where they matched best, and the D4P was re‐setup. Figure 2 shows the procedure of D4D measurement. As D4D is a fluence measuring transmission detector, the dose is measured by synthesizing the dose as measured by D4P.^18^

#### 2.C.1 Evaluation of reproducibility

As D4D is a gantry‐mounted transmission detector, the measurement value may vary depending on the mounting position. First, the reproducibility of the measurement due to the accuracy of the detector's attachment to the gantry is evaluated. D4D then obtains the measurement results based on the reference measurements collected at the same time as D4P. Thereby, we evaluate the reproducibility of the D4P setup.

##### Evaluation of D4D reproducibility in the same setup (D4D mounting reproducibility)

The D4D mounting accuracy reproducibility was evaluated for one prostate cancer treatment. First, D4P was set up to measure the dose distribution of the original plan and six simulated plans. Next, as shown in Figure 2, the reference measurements were performed using D4P and D4D. Further, using D4D only, we have measured the dose distribution of the original plan and the six types of simulated plans. The measurements in this study were performed first, with D4P alone; then, D4D was mounted and measured. This is defined as one set of measurement. D4D was dismounted and mounted before each set of measurements. To measure the D4D mounting repeatability, the mount‐dismount process was repeated ten times.

##### Evaluation of reproducibility of D4P setup (Reproducibility of reference measurement)

To evaluate the reproducibility of the D4P setup, we have used the original plan for one prostate treatment used in the evaluation of D4D reproducibility, and six types of simulated plans. D4P and D4D were used to re‐setup ten times on another day instead of the same day, and the dose distribution was measured.

#### 2.C.2 Evaluation of VMAT for ten prostate cancer patients

The setup of ten patients’ prostate treatment plans was measured similar to the evaluation of the reproducibility of the D4D setup. The process in Figure. 2 was repeated for each case and the dose distribution of D4P and D4D was measured once.

#### 2.C.3 Evaluation criteria

To evaluate the methods of 2.C.1‐2.C.2, dose deviation (DD), distance‐to‐agreement (DTA), and gamma index analysis (GA) were used for D4P and D4D reproducibility. The evaluation criteria observed were DD at 3%, DTA at 2 mm, GA at 3 %/2 mm, with the threshold set at 10%.^4^ The variation was evaluated at a 95% confidence interval (1.96 standard deviations). The GA was performed with a global normalization in the absolute doses. For the evaluation of VMAT for ten prostate cancer patients, we have performed a statistical test for significant differences between the original and simulated plans, using Welch's t‐test. A *p*‐value of 0.05 was used; after performing Bonferroni correction considering multiple comparisons, a *p*‐value less than 0.007 was considered statistically significant.

### 2.D Variation in MLC stop position and GA of prostate VMAT clinical data using D4D

Rangel *et al*. reported a less significant impact on delivery errors owing to random errors compared to systematic errors in MLC.^19^ Therefore, the evaluation of the basic error detection capability in this study was performed under the assumption that systematic errors occurred due to leaf offset placement errors during MLC adjustment. However, it is also important to investigate the MLC random errors. Therefore, we compared the deviations between the planned and measured values of the MLC stop positions A‐side and B‐side of the 2‐arc plan in five clinical cases of prostate VMAT and measured the GA values. The number of treatments in one case ranged from 39 to 40; a total of 396 A‐side and B‐side MLC stop positions were recorded. The A‐side and B‐side deviations between the average of the MLC stop positions in the treatment plan and the average of the MLC stop position measured at each treatment were calculated. The GA value was 3%/2 mm, and the threshold was 10%. The GA was performed with a global normalization in the absolute doses. The average value and standard deviation (1SD) were calculated from the average value for each 1‐arc plan.

## 3 RESULTS

### 3.A Evaluation of D4D reproducibility in the same setup (D4D mounting reproducibility)

**Fig. 3:**
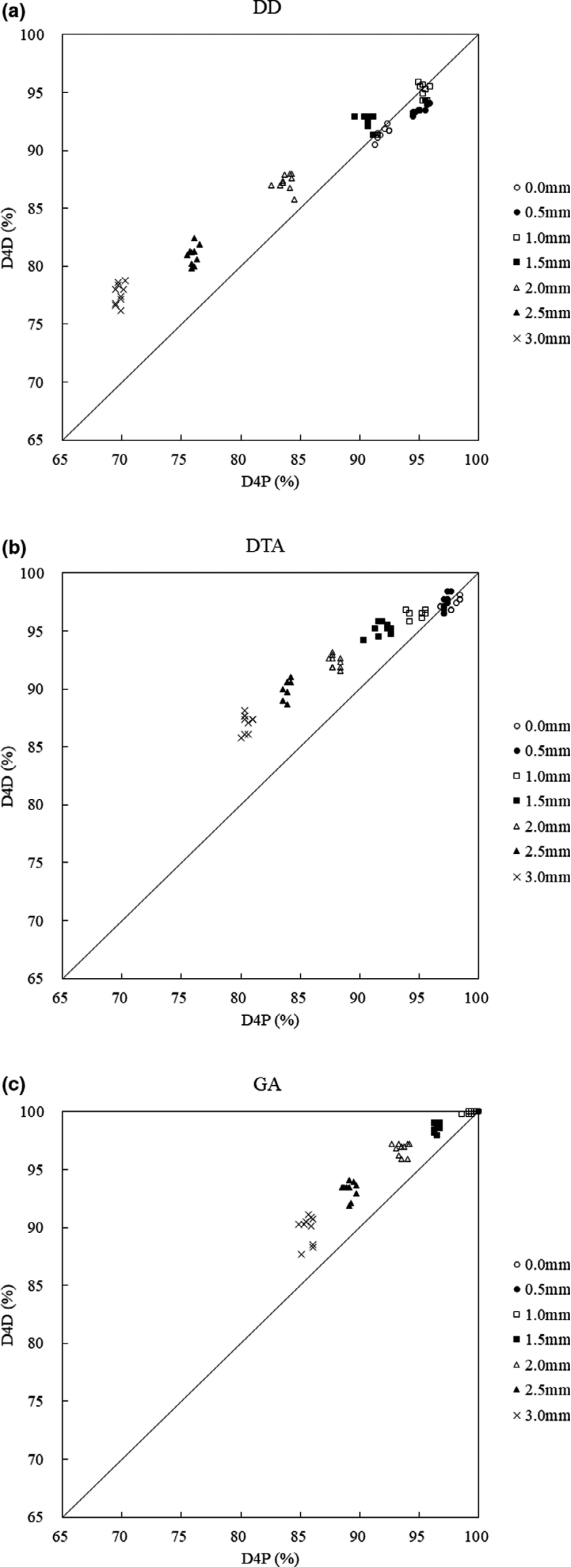
Evaluation of D4D reproducibility in the same setup (D4D mounting reproducibility). (a) DD: the horizontal axis shows the D4P pass‐ratio, and the vertical axis shows the D4D pass‐ratio. The line connecting the values where D4P and D4D are equal is defined as the reference line. (b) DTA: the horizontal axis shows the D4P pass‐ratio, and the vertical axis shows the D4D pass‐ratio. (c) GA: the horizontal axis shows the D4P pass‐ratio, and the vertical axis shows the D4D pass‐ratio.

Figure 3 shows the pass rates of DD, DTA, and GA obtained by measuring the dose distribution of the original plan and each simulated plan, repeated ten times for an evaluation of the repeatability of the D4D in the same setup.

#### 3.A.1 DD

Figure 3a shows that, when the MLC position error exceeds 1.5 mm, the D4D DD pass‐ratio exceeds that of D4P, and this tendency increases as the MLC position error increases. The maximum pass‐ratio of D4P and D4D was 95.4% and 95.0%, respectively, when the MLC position error was 1.0 mm. The minimum pass‐ratio of D4P and D4D was 69.8% and 77.6% at 3.0 mm, respectively, and the D4D minimum pass‐ratio was 7.8% higher than that for D4P. The average values of the pass‐ratio variation of the original plan and the MLC position error simulation plan were ± 0.8% for D4P and ± 1.3% for D4D. Evidently, the variation was smaller for D4P compared to that for D4D.

#### 3.A.2 DTA

Figure 3b shows that, when the MLC position error exceeds 0.5 mm, the value of D4D becomes higher than of D4P, and the tendency is similar to that of DD. The maximum pass‐ratio was 97.7% of the original plan in the case of D4P and 97.5% in the case of MLC position error of 0.5 mm in the case of D4D. The minimum pass‐ratio of D4P and D4D was 80.5% and 87.1%, respectively, at 3.0 mm, and the D4D minimum pass‐ratio was 6.6% higher than that for D4P. The average values of the pass‐ratio variation of the original plan and the MLC position error simulation plan were ± 0.9% for D4P and ± 1.2% for D4D. Evidently, the variation was smaller for D4P compared to that for D4D.

#### 3.A.3 GA

Figure 3c shows that, when the MLC position error exceeds 1.0 mm, the value of D4D becomes higher than of D4P, similar to DD and DTA. The maximum pass‐ratio of D4P and D4D was 100.0% when the original plan and the MLC position error were 0.5 mm. The minimum pass‐ratio of D4P and D4D was 85.7% and 89.8%, respectively, at 3.0 mm, and the D4D minimum pass‐ratio was 4.2% higher than that for D4P. The average values of the pass‐ratio variation of the original plan and the MLC position error simulation plan were ± 0.5% for D4P and ± 0.8% for D4D. Again, the variation was evidently smaller for D4P compared to that for D4D.

### 3.B Evaluation of reproducibility of D4P setup (Reproducibility of reference measurement)

**Fig. 4:**
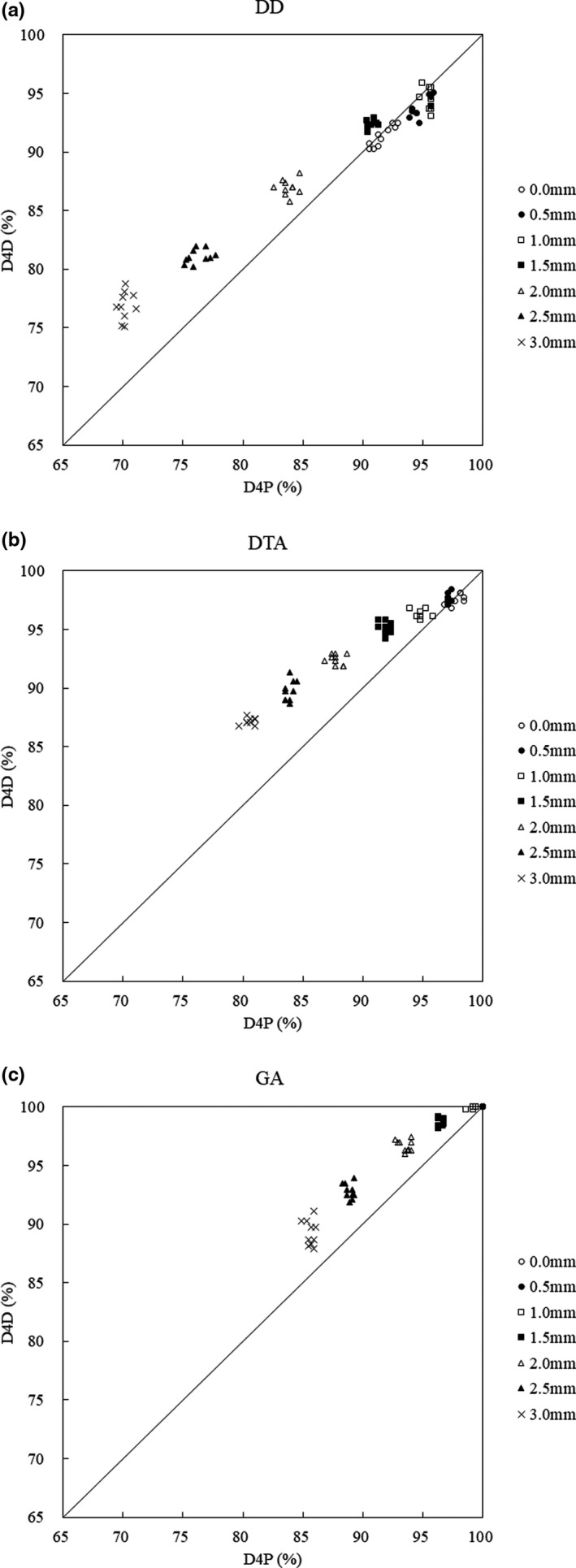
Evaluation of reproducibility of D4P setup (Reproducibility of reference measurement). (a) DD: the horizontal axis shows the D4P pass‐ratio, and the vertical axis shows the D4D pass‐ratio. The line connecting the values where D4P and D4D are equal is defined as the reference line. (b) DTA: the horizontal axis shows the D4P pass‐ratio, and the vertical axis shows the D4D pass‐ratio. (c) GA: the horizontal axis shows the D4P pass‐ratio, and the vertical axis shows the D4D pass‐ratio.

Figure 4 shows the DD, DTA and GA pass‐ratios of evaluation of the reproducibility of the D4P setup. All these pass‐ratios indicated a similar trend as evaluation of D4D reproducibility in the same setup (D4D mounting reproducibility). The differences from the evaluation of D4D reproducibility in the same setup (D4D mounting reproducibility) are shown below.

#### 3.B.1 DD

Figure 4a shows that the D4D DD pass‐ratio exceeds that of D4P when the MLC position error exceeds 1.5 mm. The maximum pass‐ratios of D4P and D4D were 95.5 and 94.6%, respectively, when the MLC position error was 1.0 mm. The minimum pass‐ratios of D4P and D4D were 70.2% and 77.9% at 3.0 mm, respectively, and the minimum pass‐ratio for D4D was 6.7% higher than that for D4P. The average values of the pass‐ratio variation of the original plan and the MLC position error simulation plan were ± 1.2% for D4P and ± 1.5% for D4D. Evidently, the variation for D4P was smaller than that for D4D. Compared to the evaluation of D4D reproducibility in the same setup (D4D mounting reproducibility), the minimum pass‐ratio for D4P became higher, the maximum pass‐ratio for D4D was 94.6%, and the minimum pass‐ratio was smaller at 76.9%. In addition, both D4P and D4D showed higher variability.

#### 3.B.2 DTA

Figure 4b shows that the value of D4D becomes higher than that of D4P when the MLC position error exceeds 0.5 mm, and the tendency is similar to that of DD. The maximum pass‐ratio was 97.7% of the original plan for D4P and 97.6% for MLC position error of 0.5 mm in the case of D4D. The minimum pass‐ratios for D4P and D4D were 80.6% and 87.2%, respectively, at 3.0 mm, and the minimum pass‐ratio for D4D was 6.6% higher than that for D4P. The average values of the pass‐ratio variation of the original plan and the MLC position error simulation plan were ±0.8% for D4P and ±0.9% for D4D. The variation for D4P was smaller than that for D4D. Compared to the evaluation of D4D reproducibility in the same setup (D4D mounting reproducibility), the variability of both D4P and D4D was small.

#### 3.B.3 GA

Figure 4c shows that the value of D4D exceeds that of D4P when the MLC position error exceeds 1.0 mm, similar to DD and DTA. The maximum pass‐ratio for D4P and D4D was 100.0% when the original plan and the MLC position error were 0.5 mm. The minimum pass‐ratios for D4P and D4D were 85.6% and 89.3%, respectively, at 3.0 mm, and the minimum pass‐ratio for D4D was 3.7% higher than that for D4P. The average values of the pass‐ratio variation of the original plan and the MLC position error simulation plan were ± 0.4% for D4P and ± 0.8% for D4D. Similarly, the variation for D4P was evidently smaller than that for D4D. Compared to the evaluation of D4D reproducibility in the same setup (D4D mounting reproducibility), the minimum pass‐ratio of D4D became smaller. Additionally, the variation was almost the same for both D4P and D4D.

### 3.C Evaluation of VMAT for ten prostate cancer patients

**Fig. 5:**
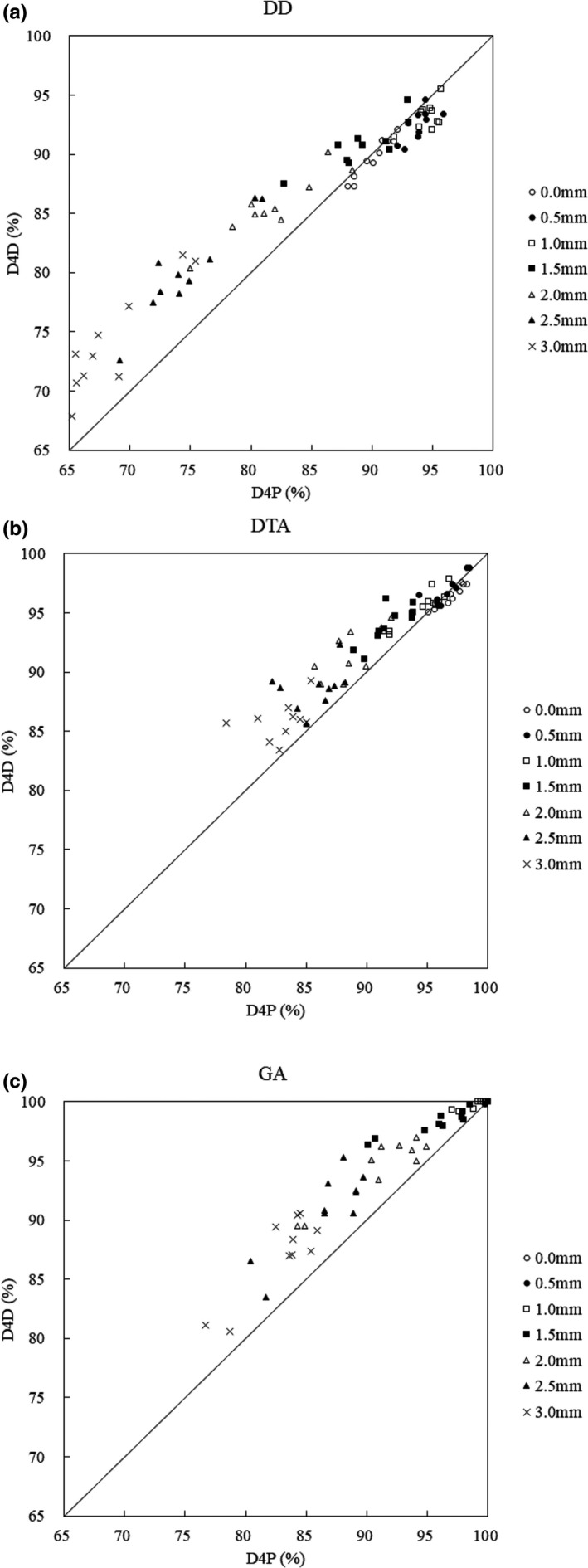
Evaluation of VMAT for ten prostate cancer patients. (a) DD: the horizontal axis shows the D4P pass‐ratio, and the vertical axis shows the D4D pass‐ratio. The line connecting the values where D4P and D4D are equal is defined as the reference line. (b) DTA: the horizontal axis shows the D4P pass‐ratio, and the vertical axis shows the D4D pass‐ratio. (c) GA: the horizontal axis shows the D4P pass‐ratio, and the vertical axis shows the D4D pass‐ratio.

The DD, DTA, and GA pass‐ratios of evaluation of VMAT for ten prostate cancer patients are shown in Figure 5. These pass‐ratios tend to be smaller than the evaluation of D4D reproducibility in the same setup (D4D mounting reproducibility) and the evaluation of the reproducibility of D4P setup (Reproducibility of reference measurement). Additionally, the variability was higher for both D4P and D4D. The differences from the evaluation of D4D reproducibility in the same setup (D4D mounting reproducibility) and evaluation of the reproducibility of D4P setup (Reproducibility of reference measurement) are shown below.

#### 3.C.1 DD

Figure 5a shows that the pass‐ratio of D4D DD exceeds that of D4P when the MLC position error exceeds 1.5 mm, and this tendency increases as the MLC position error increases. When the MLC position error was 1.0 mm, the maximum pass‐ratios for D4P and D4D were 94.5 and 93.2% respectively. The minimum pass‐ratios of D4P and D4D were 68.6% and 74.2% at 3.0 mm, respectively, and the minimum pass‐ratio for D4D was 5.6% higher than that for D4P. The average values of the pass‐ratio variation of the original plan and the MLC position error simulation plan were ±5.0% for D4P and ±4.9% for D4D. The variation for D4D was smaller than that for D4P. The maximum and minimum pass‐ratio D4P and D4D were smaller than the evaluation of D4D reproducibility in the same setup (D4D mounting reproducibility) and evaluation of reproducibility of D4P setup (Reproducibility of reference measurement). In addition, both D4P and D4D showed higher variability.

#### 3.C.2 DTA

Figure 5b shows that the value for D4D was higher than that for D4P when the MLC position error exceeded 0.5 mm, and the tendency was similar to that of DD. The maximum pass‐ratio was 96.9% of the original plan for D4P and 96.8% for MLC position error of 0.5 mm for D4D. The minimum pass‐ratios for D4P and D4D were 83.0% and 85.9%, respectively, at 3.0 mm, and the D4D minimum pass‐ratio was 2.9% higher than that for D4P. The average values of the pass‐ratio variation of the original plan and the MLC position error simulation plan were ±3.4% for D4P and ±3.0% for D4D. The variation for D4D was smaller than that for D4P. The minimum pass‐ratio for D4P was greater than evaluation of D4D reproducibility in the same setup (D4D mounting reproducibility) and evaluation of the reproducibility of D4P setup (Reproducibility of reference measurement).

#### 3.C.3 GA

Figure 5c shows that the value for D4D was higher than that for D4P when the MLC position error exceeded 1.0 mm, similar to DD and DTA. The maximum pass‐ratio for D4P and D4D was 100.0% when the original plan and the MLC position error were 0.5 mm. The minimum pass‐ratios for D4P and D4D were 82.9% and 87.1%, respectively, at 3.0 mm, and the minimum pass‐ratio for D4D was 4.2% higher than that for D4P. The average values of the pass‐ratio variation of the original plan and the MLC position error simulation plan were ±3.9% for D4P and ± 3.2% for D4D. Similarly, the variation for D4D was evidently smaller than that for D4P. The maximum pass‐ratios were the same for both D4P and D4D. However, variation was observed at 0.5 mm.

#### 3.C.4 Statistical significance test

We performed a statistical significance test to evaluate the differences between the simulated plan and the original D4D plan using Welch's t‐test on DD, DTA, and GA. Table 1 lists the *p*‐values. The D4D *p*‐value for comparing DD was *p* = 0.194 when the MLC position error was 1.5 mm; the *p*‐value of DTA for the MLC position errors of 0.5 mm and 1.0 mm were *p* = 0.397 and *p* = 0.175, respectively; and the *p*‐value of GA with the MLC position error at 0.5 mm and 1.0 mm, showed no significant differences between the *p* = 0.168 and *p* = 0.084 values. When the positional error of MLC was 0.5 and 1.0 mm, the pass rate with DD was higher than that in the original plan, with a significant difference (*p* < 0.007). When the positional error of MLC was 2.0, 2.5, and 3.0 mm, the pass rate with DD was lower than that in the original plan, with a significant difference (*p* < 0.007). DTA and GA showed a significant difference at *p* < 0.007 when the MLC position error from the original plan increased from 1.5 mm. When DD, DTA and GA parameters were used, the detection of MLC error was 2.0 mm for DD and 1.5 mm for DTA and GA.

**Table 1:** Comparison of *p*‐value that all MLC position error simulation plans can detect in ten plans.

Evaluation method	Device	MLC error (mm)						
		0.0	0.5	1.0	1.5	2.0	2.5	3.0
DD	D4P	1.000	<0.001*	<0.001*	0.419	<0.001*	<0.001*	<0.001*
	D4D	1.000	<0.001*	<0.001*	0.194	0.001*	<0.001*	<0.001*
DTA	D4P	1.000	0.528	0.003*	<0.001*	<0.001*	<0.001*	<0.001*
	D4D	1.000	0.397	0.175	0.001*	<0.001*	<0.001*	<0.001*
GA	D4P	‐	0.168	0.018	0.001*	<0.001*	<0.001*	<0.001*
	D4D	‐	0.168	0.084	<0.001*	<0.001*	<0.001*	<0.001*

Statistical tests were performed on the significance of the original design and the simulated design (MLC position error 0.5, 1.0, 1.5, 2.0, 2.5, 3.0 mm) using Welch's t‐test. Both D4P and D4D were evaluated. Using a Bonferroni correction to account for multiple comparisons using 0.05, a *p*‐value was considered to be statistically significant when it was less than 0.007.

* Statistically significant (*p* < 0.007), ( ‐ ) incalculable because D4P and D4D are 100%.

Furthermore, the statistical test was performed for D4P. For D4P, MLC error detection was 2.0 mm for DD, 1.0 mm for DTA, and 1.5 mm for GA.

### 3.D Variation in MLC Stop Position and GA of Prostate VMAT Clinical Data Using D4D

According to the data listed in Table 2, the variation of MLC stopping position of prostate VMAT (A‐side and B‐side) was calculated. The deviation from the measured value was stable at an average of −0.09 ± 0.05 mm (maximum +0.17 ± 0.07 mm, minimum −0.46 ± 0.03 mm) in all five cases, and all the GA values were above 99.86%. The variation of the GA value was as small as ±0.11%.

**Table 2:** Variation in MLC Stop Position and GA of Prostate VMAT Clinical Data Using D4D.

Patient	Arc number	Side	MLC error (mm)	Gamma analysis (%)
Patient1	Arc 1	A side	0.04 ± 0.04	99.98 ± 0.08
		B side	−0.25 ± 0.05	
	Arc 2	A side	−0.07 ± 0.06	99.98 ± 0.11
		B side	−0.31 ± 0.03	
Patient2	Arc 1	A side	0.10 ± 0.05	99.86 ± 0.25
		B side	−0.29 ± 0.07	
	Arc 2	A side	−0.09 ± 0.04	99.93 ± 0.30
		B side	−0.46 ± 0.03	
Patient3	Arc 1	A side	0.15 ± 0.06	99.98 ± 0.06
		B side	−0.07 ± 0.05	
	Arc 2	A side	0.17 ± 0.07	99.94 ± 0.12
		B side	−0.10 ± 0.05	
Patient4	Arc 1	A side	0.10 ± 0.05	99.94 ± 0.09
		B side	−0.08 ± 0.05	
	Arc 2	A side	0.02 ± 0.06	100.00 ± 0.00
		B side	−0.12 ± 0.07	
Patient5	Arc 1	A side	−0.08 ± 0.03	99.93 ± 0.10
		B side	−0.26 ± 0.05	
	Arc 2	A side	−0.03 ± 0.05	100.00 ± 0.00
		B side	−0.22 ± 0.04	

The average and 1SD of MLC stop position variation (A‐Side and B‐Side) of prostate VMAT clinical data by D4D were calculated. The average and 1SD of the GA of each 1arc plan were calculated.

## 4 DISCUSSION

In this study, we evaluated the ability of the D4D for detecting MLC position error, measuring the dose distribution of the original plan and simulated plan. As EPID in a previous study reportedly detected an MLC error of 3 mm during treatment, we evaluated whether the gantry‐mounted transmission detector used in this study had the same order of detection capability.^20^, ^21^ First, we assessed the reproducibility of the D4D setup for the same day. To ensure the output stability, the output coefficient of variation of the output measurement of weekly QA during the data collection period was calculated. The variation coefficient of the output dose of the linear‐accelerator device used in this study was found to be stable at 0.19% in the weekly QA dose control using an ionization chamber. The one daily short‐term reproducibility output coefficient of variation was 0.01%. In the previous reports, the short‐term reproducibility of ten D4P measurements was 0.1% (1SD) and the long‐term stability was 0.5% (1SD).^17^ For DD, DTA, and GA measured by D4D, the variation in the pass‐ratio displayed a tendency to increase as the MLC position error increased. However, the variation in the pass‐ratio for DD, DTA, and GA of D4P for each plan did not change significantly. The possible explanation of these results could be the uncertainties in the setup of the D4P and D4D dose verification systems, and the uncertainties of the D4D and D4P system mechanical features. Since D4D was mounted and dismounted for each measurement, the uncertainties due to the D4D mounting/demounting were a potential reason for the setup error. Next, we considered the uncertainty due to the mechanical accuracy of the D4D and D4P systems. Regarding the mechanical accuracy of the D4D and D4P systems, the difference in the geometric arrangement between D4D and D4P could induce the difference in the detector arrangement, and affect the rotation of the collimator. In D4D, detectors are mounted on the gantry head, therefore, rotated along with the collimator. In contrast, detectors of D4P, as placed on the treatment couch, do not rotate with the rotation of the collimator. Further, rotating the collimator may affect the D4D and D4P dose verification results. In addition, the effect of the detector spacing is considered. The D4P detectors are placed at a distance of 5.0 mm between the elements, within a 60 mm × 60 mm area at the center of each board, and 10.0 mm between the elements outside the center area of 60 mm × 60 mm. The D4D detectors are arranged at intervals of 2.5 mm and 5.0 mm in the X and Y directions, respectively, in terms of the isocenter. As a result, D4D exhibits a better resolution than D4P. The display of D4D dose distribution shows the dose changes at the isocenter, not on the detector surface of D4D. These complex factors such as detector spacing, detector surface, and isocenter surface dose distribution affect D4D dose distribution. The D4P provides the point dose difference in a 3D position around the isocenter, whereas the D4D shows the errors in 2D fluence. D4P shows the measurement results of a single unit. As D4D of a single unit cannot be measured, a dose distribution based on the measurement results of the original D4P plan is used to compute D4D. Therefore, the measurement results of D4D are probably affected by the uncertainty of the measurement results of D4P. Additionally, since the dose distribution of D4D is obtained by calculations based on the MLC position error of 0 mm of the D4P original plan, the error was amplified as the MLC position error increased, so the variation of the pass‐ratio was expected to increase.

We now explain why the pass rate of the MLC position error of 0.5 mm was higher than the original plan of DD. The setup of the D4P in this study comprised an irradiation field created by 10 cm × 10 cm jaws, and the dose profile was confirmed by the software attached to the D4P. Based on the profile calculated by the TPS, the treatment couch was moved to the position where the two profiles matched best by 0.1 mm, and the D4P was re‐setup. However, we believe that there was a small difference between the center position of the MLC and the jaws, as well as between the sagging of the gantry and the shift of the gantry rotation center. It is considered that these factors were complicatedly interconnected; thus, the pass rate of the MLC position error of 0.5 mm was higher than that of the original plan. The MLC position error plan in this study is the one in which the MLC stop position was systematically moved at all MLC control points. Therefore, the entire dose distribution shifted systematically. In particular, when the steep dose gradient area shifted systematically, this induced a significant effect on DD. It is conceivable that DD overestimated the error because it shows a considerable dose difference with a small positional error in the area where the dose gradient is steep. Because DTA is useful for detecting displacement in areas with steep dose gradients, we consider that the effect of the difference in the pass‐ratio between the original plan and the plan simulated MLC position error was small. Next, the effect of the stop position error of MLC was not detected because GA is a parameter that simultaneously detects the dose difference, and the position error.^22^

Next, the evaluation of reproducibility of the D4P setup is considered. In addition to the evaluation of D4D reproducibility in the same setup, the re‐setup of D4P also has an effect. The DD of D4P variation with a 95% confidence interval (1.96 standard deviations), the evaluation of the setup reproducibility of the reference D4P data was ±0.7% to ±1.7%. This value showed an increase in the variation due to the D4P setup error in the evaluation of the D4D setup reproducibility, on the same day. DTA and GA showed the same tendency as DD concerning the variation due to the D4P setup error. The evaluation of D4D reproducibility in the same setup, and the evaluation of setup reproducibility of D4P data as a reference, are important items of reproducibility when introducing D4P and D4D as reported by Li G. et al.^23^

Finally, we considered the evaluation of VMAT in ten patients with prostate cancer. A Welch’s t‐test for the original plan and each simulated plan in D4D indicated that DD showed no significant difference when the MLC position error deviated by 1.5 mm from the original plan. This is probably because the MLC position error of 1.5 mm and the average value of the original plan DD became equal. DD showed a significant difference when the MLC position error was 2.0 mm or more. However, DTA and GA, parameters for evaluating position information, showed significant differences with MLC position errors of 1.5 mm or more. The error detection accuracy of DD is unstable in the MLC position error plan; it was suggested that errors could be detected correctly by using DTA and GA. Therefore, in clinical use, the value of DTA and GA should be regarded as more important than the value of DD. In the linear accelerator used in this study, MLC adjustment is performed periodically. During the periodic MLC adjustment, systematic errors due to leaf offset mounting position errors may lead to accidents. Therefore, we assumed that the systematic error caused by the position error of the leaf offset during the MLC adjustment occurred in this study.

Rangel *et al*. reported a less significant impact on delivery errors owing to random errors compared to systematic errors in MLC,^19^ and in the present study, the MLC stopping position during treatment between 39 and 40 days of the five prostate VMAT clinics was confirmed to be up to ± 0.07 mm at 1 SD. For random errors, it was small and stable. The GA value was 99.93 ± 0.30% at a maximum of −0.46 ± 0.03 mm, even if the amount of error was large compared to the systematic error of the MLC. Our results suggest that the variability of the GA value is reflected in the magnitude of error when the MLC systematic error is large. However, since it greatly exceeded the standard GA value of 95% stated in the AAPM guideline,^4^ it was considered that the fluctuation was within the standard preverification criteria. Therefore, the results of the five cases of prostate VMAT in the present study suggest that the impact of random errors on delivery errors is significantly less than that of systematic errors.

The UK guidelines state that error detection with the same (or better) accuracy as an EPID system can be considered useful in IVD.^13^ Liang et al. reported a minimum detectable MLC position error of 2.0 mm for D4P and 3.0 mm for EPID based on receiver operating characteristic analysis of GA 2%/2 mm for head and neck VMATs.^20^ In addition, Young et al. reported a study of MLC position error in prostate VMAT using EPID; using the combination of Elekta Synergy and in‐house software the MLC position error was 7.0 mm to below the GA of 3%/3 mm 90%, and the MLC position error is 3.0 mm below the GA of 2%/2 mm 90%.^21^ In addition, *Arumugam et al. examined* the MLC position error of prostate VMAT using D4P, and reported that a GA standard of 2%/2 mm could detect an MLC position error of 2.0 mm or more by a significant difference test.^24^ Although it is difficult to directly compare the present study with these previous studies because the situation is different from these previous studies, the MLC position error was detectable at 1.5 mm under our limited conditions. In this study, the significant differences between the D4D and D4P with GA criteria of 3%/2 mm were 1.5 mm. However, statistical testing is not a method for pass/fail evaluation with clinical data. For clinical data pass/fail, as mentioned earlier, the recommended evaluation criteria for TG218 of the AAPM (set based on 3%/2mm at 10% dose threshold) and a certain gamma pass rate (95%) would be selected.^4^ When comparing the two detectors (D4D and D4P), it is important to determine the level of MLC bank offset at which each detector will fail the gamma ray pass rate. This study aimed to determine the level of MLC bank offset at which each of the two detectors (D4D and D4P) would lead to failure of the gamma pass rate when comparing the two detectors. D4P generally yielded failed results at lower leaf bank offset values than those of D4D, suggesting that D4D is a more sensitive device. Assuming a 95% pass rate, some plans passed with D4D but failed with D4P at leaf offsets of 1.5 and 2 mm. The user should determine whether D4D is sensitive enough for general use after evaluating the data based relevant guidelines. The UK guidelines do not specify the procedure to assess the accuracy of the system or the indicators that must be used.^13^ Therefore, we believe that this study contains useful information for D4D and D4P users. Furthermore, MLC position error can be detected by D4D alone, and we can expect its application in the detection of delivery errors during irradiation.

Assuming that D4D is used for the detection of delivery errors during irradiation, it is possible to accurately detect errors of the linear accelerator alone since the output dose distribution emitted from the linear accelerator is directly observed. In contrast, when EPID is used, the dose distribution of radiation penetrated through the patient is measured, the effects of body shape change, and patient setup are included. Therefore, it is challenging to evaluate error detection only for the linear accelerator alone.

A 2010 report from the Netherlands highlighted the importance of dose verification during treatment.^10^, ^11^ Among the MLC positional errors that occur during VMAT and IMRT treatment, this study shows that systematic shifts in both MLC banks can be used to detect delivery errors during irradiation at a constant level. In this study, error detection analysis was performed on the prostate as it is considered to have the least intensity modulation among the sites.^4^ However, additional studies are required for more detailed detection of errors during irradiation, involving other sites such as head and neck cases.

## 5 CONCLUSION

In this study, we examined the suitability of D4D in detecting MLC position error. It was found that D4D can detect delivery errors during irradiation. D4D has almost the same detection power as D4P and can detect MLC position errors of 1.5 mm or more using DTA or GA. In conclusion, a transmission‐type detector can be suitable for the detection of delivery errors during irradiation.

## CONFLICT OF INTEREST

The authors declare that there are no conflicts of interest.

## AUTHOR CONTRIBUTIONS

The conceptual design of the study was carried out by H. Honda, M. Tominaga, M. Sasaki and Y. Uto. Data were collected by Y. Hamamoto, Y. Ishii, R. Yamamoto, T. Mochizuki and T. Kido. Data analysis was performed by H. Honda, M. Oita, and H. Kanzaki. Interpretation of the submitted papers was discussed by all authors. All authors wrote or critically revised their articles on important intellectual content. In addition, all authors have given final approval to the submitted papers.

## Data Availability

The data that support the findings in this study are from cases conducted at our hospital. Also, the database released for other institutions is not subject to ethical review approval. Furthermore, due to privacy and ethical restrictions, data provision is not available upon request. Therefore, data sharing is not applicable.
